# Supplementary material for: The Role of Oxidative Stress Markers in Predicting Acute Thrombotic Occlusion of Haemodialysis Vascular Access and Progressive Stenotic Dysfunction Demanding Angioplasty
Source: Antioxidants (Basel). 2021 Apr 8;10(4):569. doi: 10.3390/antiox10040569 (PMC8068110; doi:10.3390/antiox10040569)
Supplement: Supplementary file 1 [file antioxidants-10-00569-s001.pdf]

Table S1. Univariate and multivariate Cox regression analyses for the predictors of dysfunction of arteriovenous fistulas/grafts within 12 months of follow-up.

Parameter	Univariate analysis		Multivariate analysis	
	HR (95% Cl)	*P*	HR (95% Cl)	*p*
Age (years)	0.98 (0.95–1.02)	0.312	0.97 (0.94–1.01)	0.124
Sex		0.506		0.862
Female	1.00		1	
Male	0.76 (0. 34–1.70)		1.08 (0.44–2.66)	
Access type (AVF/AVG)				
AVF	1.00		1	
AVG	2.28 (1.03–5.08)	0.043*	2.42 (0.92–6.35)	0.073
Risk factors				
Hypertension (%)	1.65 (0.49–5.53)	0.418		
Diabetes mellitus (%)	1.36 (0.61–3.04)	0.448		
Current smoking (%)	0.99 (0.23–4.20)	0.986		
CAD (%)	1.26 (0.56–2.83)	0.581		
PAOD (%)	0.57 (0.17–1.90)	0.357		
Plasma biochemical data				
LDL-C (mg/dL)	1.01 (0.99–1.02)	0.193		
HDL-C (mg/dL)	1.00 (0.98–1.03)	0.794		
TG (mg/dL)	1.00 (0.99–1.00)	0.735		
Calcium (mg/dL)	1.44 (1.00–2.10)	0.053		
Phosphate (mg/dL)	1.14 (0.87–1.48)	0.354		
Albumin (mg/dL)	1.17 (0.35–3.86)	0.799		
Creatinine (mg/dL)	1.09 (0.94–1.26)	0.258		

Kt/V	0.71 (0.16−3.13)	0.652		
Medications				
Anti-platelet agents	1.35 (0.59−3.07)	0.482		
Aspirin	0.96 (0.43−2.14)	0.918		
Dipyridamole	1.41 (0.48−4.12)	0.532		
Cilostazol	0.77 (0.10−5.67)	0.793		
Coumadin	3.06 (0.72−13.03)	0.13		
Nitrates	1.10 (0.49−2.47)	0.821		
β-blockers	0.89 (0.38−2.07)	0.777		
Calcium antagonists	0.81 (0.36−1.80)	0.603		
ACEI/ARB	0.99 (0.41−2.38)	0.973		
Diuretic	4.23 (1.85−9.66)	0.001*	1.80 (0.62−5.16)	0.277
Lipid-lowering agents				
Statin	2.80 (1.05−7.50)	0.040*	1.16 (0.35−3.85)	0.810
Fibrate	0.05 (<0.0001−3221.32)	0.592		
Plasma biomarkers				
Hs-CRP (mg/dL)	1.01 (0.83−1.42)	0.907		
Homocysteine (μmol/L)	0.99 (0.95−1.03)	0.634		
**ADMA >0.6207 (μmol/mL)**	**7.20 (2.44−21.30)**	**<0.001***	**4.55 (1.20−17.26)**	**0.026***
MMP-2 (ng/mL)	0.999 (0.997−1.001)	0.471		
MMP-9 (ng/mL)	1.01 (1.00−1.02)	0.011*	1.00 (0.99−1.01)	0.712
NO (μM)	1.00 (1.000−1.002)	0.089		
MCP-1 (pg/mL)	1.00 (0.997−1.002)	0.711		
TGF-β (pg/mL)	1.01 (0.96−1.06)	0.860		
TNF-α (pg/mL)	0.99 (0.98−1.00)	0.204		

IL-1β (pg/mL)	1.01 (0.999–1.015)	0.086

*: p-value<0.05; multivariate analysis: adjusted variables with significance ($p < 0.05$) in univariate analysis. HR was presented with 95% CI (range).

Abbreviations: AVF, arteriovenous fistula; AVG, arteriovenous graft; Hs-CRP, high-sensitivity C-reactive protein; LDL-C, low-density lipoprotein cholesterol; MMP-2, matrix metalloproteinase-2; MMP-9, matrix metalloproteinase-9; NO, nitrate oxidase; PAOD, peripheral arterial occlusive disease; TG, triglyceride; IL-1β: interleukin-1β; MCP-1: monocyte chemotactic protein 1; TNF-α: tumour necrosis factor-α; TGF-β: transforming growth factor-β.

Table S2. Oxidative stress markers and acute thrombosis stratified by vascular access type.

Parameter	AVF			AVG		
	Acute thrombosis (n = 3)	Non-thrombosis (n = 103)	p-value	Acute thrombosis (n = 21)	Non-thrombosis (n = 32)	p-value
hsCRP (mg/dL)	0.18 ± 0.18	1.11 ± 2.19	0.355	1.05 ± 1.19	1.28 ± 1.92	0.723
Homocysteine (μmol/L)	56.02 ± 27.21	27.65 ± 11.35	0.008*	26.27 ± 8.29	24.56 ± 6.29	0.454
ADMA (μmol/mL)	0.85 ± 0.13	0.57 ± 0.26	0.05	0.65 ± 0.28	0.62 ± 0.24	0.707
MMP2 (ng/mL)	685.00 ± 239.62	866.02 ± 211.64	0.233	771.04 ± 175.64	820.30 ± 199.51	0.304
MMP9 (ng/mL)	94.80 ± 40.89	59.36 ± 39.99	0.118	57.45 ± 40.45	72.51 ± 43.22	0.118
NO (μM)	303.37 ± 335.87	241.78 ± 221.86	0.992	207.40 ± 178.34	306.97 ± 471.86	0.422
MCP-1 (pg/mL)	454.19 ± 75.12	334.46 ± 183.16	0.058	311.73 ± 125.45	345.39 ± 162.36	0.366
TGF-β (pg/mL)	7.70 ± 6.86	9.20 ± 9.43	0.902	4.19 ± 4.36	4.91 ± 4.12	0.348
TNF-α (pg/mL)	23.48 ± NA	76.78 ± 106.09	0.788	69.99 ± 118.89	40.00 ± 70.95	0.494
IL-1β (pg/mL)	102.26 ± 48.68	51.33 ± 38.06	0.068	67.48 ± 38.71	73.81 ± 45.99	0.809

Acute thrombosis of AVF				
Independent variable	univariate analysis<br>HR (95% CI)	*p*-value	multivariate analysis<br>HR (95% CI)	*p*-value
Homocysteine	1.08 (1.03–1.14)	0.002*	1.08 (1.03–1.15)	0.004*
ADMA	22.54 (0.82–618.32)	0.065	39.81 (0.46–3441.08)	0.105

*: *p*-value<0.05; multivariate analysis: adjusted variables with significance ($p<0.05$) in univariate analysis. HR was presented with 95% CI (range).

Abbreviations: AVF, arteriovenous fistula; AVG, arteriovenous graft; Hs-CRP, high-sensitivity C-reactive protein; LDL-C, low-density lipoprotein cholesterol; MMP-2, matrix metalloproteinase-2; MMP-9, matrix metalloproteinase-9; NO, nitrate oxidase; PAOD, peripheral arterial occlusive disease; TG, triglyceride; IL-1β: interleukin-1β; MCP-1: monocyte chemotactic protein 1; TNF-α: tumour necrosis factor-α; TGF-β: transforming growth factor-β.

Table S3. Oxidative stress markers and dysfunction stratified by vascular access type.

Parameter	AVF			AVG		
	Dysfunction (*n* = 12)	Non-Dysfunction (*n* = 94)	*p*-value	Dysfunction (*n* = 12)	Non-Dysfunction (*n* = 41)	*p*-value
hsCRP (mg/dL)	1.15 ± 2.26	1.08 ± 2.16	0.333	1.03 ± 1.16	1.24 ± 1.79	0.823
Homocysteine (μmol/L)	28.20 ± 6.14	28.49 ± 13.34	0.414	24.34 ± 6.60	25.49 ± 7.32	0.637
ADMA (μmol/mL)	0.82 ± 0.33	0.55 ± 0.23	0.007*	0.73 ± 0.20	0.60 ± 0.26	0.111
MMP2 (ng/mL)	845.29 ± 237.34	862.67 ± 211.70	0.846	746.28 ± 203.67	816.73 ± 185.70	0.372
MMP9 (ng/mL)	82.00 ± 43.01	57.83 ± 39.38	0.064	81.75 ± 52.60	62.09 ± 38.57	0.21
NO (μM)	252.67 ± 123.99	242.35 ± 233.88	0.202	463.00 ± 719.22	210.30 ± 185.82	0.13
MCP-1 (pg/mL)	340.27 ± 108.55	337.81 ± 189.44	0.429	323.05 ± 99.99	333.83 ± 160.12	0.594
TGF-β (pg/mL)	11.33 ± 8.35	8.87 ± 9.44	0.21	5.16 ± 3.06	4.45 ± 4.54	0.283
TNF-α (pg/mL)	21.28 ± 16.66	82.16 ± 109.53	0.153	6.27 ± 3.21	63.24 ± 95.50	0.053
IL-1β (pg/mL)	70.04 ± 35.51	50.72 ± 39.20	0.059	80.85 ± 44.74	68.00 ± 42.24	0.421

Dysfunction of AVF		
Independent variable	HR (95% CI)	*p*-value
ADMA	28.93 (3.94–212.58)	0.001*

*: *p*-value<0.05; multivariate analysis: adjusted variables with significance ($p < 0.05$) in univariate analysis. HR was presented with 95% CI (range).

Abbreviations: AVF, arteriovenous fistula; AVG, arteriovenous graft; Hs-CRP, high-sensitivity C-reactive protein; LDL-C, low-density lipoprotein cholesterol; MMP-2, matrix metalloproteinase-2; MMP-9, matrix metalloproteinase-9; NO, nitrate oxidase; PAOD, peripheral arterial occlusive disease; TG, triglyceride; IL-1β: interleukin-1β; MCP-1: monocyte chemotactic protein 1; TNF-α: tumour necrosis factor-α; TGF-β: transforming growth factor-β.

Table S4. Correlation matrix between clinical factors and oxidative stress markers (Pearson's correlation statistics).

	Acute.thrombosis	Dysfunction	hsCRP	Homocysteine	ADMA	MMP2	MMP9	NO	MCP.1	TGF.β	TNF.α	IL.1β
Acute.thrombosis	0	0.02	0.63	0.22	0.11	0.04	0.96	0.55	0.87	0.13	0.88	0.09
Dysfunction	0.02	0	0.93	0.6	0	0.24	0.01	0.05	0.9	0.85	0.05	0.04
hsCRP	0.63	0.93	0	0.16	0.53	0.59	0.01	0.07	0.83	0.82	0.84	0.1
Homocysteine	0.22	0.6	0.16	0	0	0.24	0.01	0.54	0.96	0.09	0.77	0.02
ADMA	0.11	0	0.53	0	0	0.16	0	0.68	0.91	0.13	0.15	0
MMP2	0.04	0.24	0.59	0.24	0.16	0	0.13	0.72	0.46	0.04	0.77	0.05
MMP9	0.96	0.01	0.01	0.01	0	0.13	0	0.64	0.59	0.52	0.23	0
NO	0.55	0.05	0.07	0.54	0.68	0.72	0.64	0	0.56	0.73	0.28	0.16
MCP.1	0.87	0.9	0.83	0.96	0.91	0.46	0.59	0.56	0	0.79	0.03	0.11
TGF.β	0.13	0.85	0.82	0.09	0.13	0.04	0.52	0.73	0.79	0	0.38	0.85
TNF.α	0.88	0.05	0.84	0.77	0.15	0.77	0.23	0.28	0.03	0.38	0	0.02
IL.1β	0.09	0.04	0.1	0.02	0	0.05	0	0.16	0.11	0.85	0.02	0

Abbreviations: AVF, arteriovenous fistula; AVG, arteriovenous graft; Hs-CRP, high-sensitivity C-reactive protein; LDL-C, low-density lipoprotein cholesterol; MMP-2, matrix metalloproteinase-2; MMP-9, matrix metalloproteinase-9; NO, nitrate oxidase; PAOD, peripheral arterial occlusive disease; TG, triglyceride; IL-1β: interleukin-1β; MCP-1: monocyte chemotactic protein 1; TNF-α: tumour necrosis factor-α; TGF-β: transforming growth factor-β.
